# Comparison between phase feeding and diet blending on growth performance, carcass characteristics, and diet economics of finishing pigs raised in a commercial environment

**DOI:** 10.1093/tas/txag027

**Published:** 2026-02-28

**Authors:** Ron Aldwin S Navales, Mike D Tokach, Mike E Reard, Alan J Warner, Chad Hastad, Joel M DeRouchey, Katelyn N Gaffield, Jordan T Gebhardt, Robert D Goodband, Jason C Woodworth

**Affiliations:** Department of Animal Sciences and Industry, College of Agriculture, Kansas State University, Manhattan, KS 66506-0201, United States; Department of Animal Sciences and Industry, College of Agriculture, Kansas State University, Manhattan, KS 66506-0201, United States; ComDel Innovation, Wahpeton, ND 58075, United States; New Fashion Pork, Jackson, MN 56143, United States; New Fashion Pork, Jackson, MN 56143, United States; Department of Animal Sciences and Industry, College of Agriculture, Kansas State University, Manhattan, KS 66506-0201, United States; Department of Animal Sciences and Industry, College of Agriculture, Kansas State University, Manhattan, KS 66506-0201, United States; Department of Diagnostic Medicine/Pathobiology, College of Veterinary Medicine, Kansas State University, Manhattan, KS 66506-0201, United States; Department of Animal Sciences and Industry, College of Agriculture, Kansas State University, Manhattan, KS 66506-0201, United States; Department of Animal Sciences and Industry, College of Agriculture, Kansas State University, Manhattan, KS 66506-0201, United States

**Keywords:** diet blending, growing-finishing pigs, phase feeding, standardized ileal digestible Lys

## Abstract

A total of 2160 pigs (initially 24.8 ± 0.97 kg) and 962 pigs (initially 26.5 ± 0.37 kg) were used in 2 experiments to compare diet blending with phase feeding on growth performance and profitability. Pigs were housed in mixed-gender pens with 20 and 26 pens per treatment in Exp. 1 and 2, respectively. Pens were assigned to treatment in a randomized complete block design and blocked by initial body weight. In Exp. 1, pens were assigned to treatments arranged in a 2 × 2 factorial comparing feeding strategy (phase feeding vs. diet blending) and standardized ileal digestible **(SID)** Lys (90 or 100% of requirement estimates). Phase-fed pigs were fed diets in 5 phases. For diet blending, low and high SID Lys diets were blended daily to achieve 90 or 100% of SID Lys requirements. Overall average daily gain **(ADG)** was not influenced by feeding strategy, but diet blending decreased (*P* = 0.002) average daily feed intake **(ADFI)** and increased (*P* < 0.001) gain to feed ratio **(G:F)**. Hot carcass weight **(HCW)**, fat depth, and loin depth were not affected; however, diet blending tended to reduce (*P* = 0.074) carcass yield and increased (*P* = 0.094) percentage lean. There was a tendency for greater (*P* = 0.066) income over feed cost **(IOFC)** with phase feeding under low ingredient prices, but diet blending had lower (*P* = 0.049) feed cost/kg gain under high prices. Lysine level did not affect overall growth performance, but pigs fed the 90% SID Lys diets had lower (*P* < 0.001) feed cost. In Exp. 2, two feeding strategies were compared. Phase-fed pigs were provided with diets in 3 phases until 114 kg with a common diet thereafter. Pigs fed the diet blending strategy used 2 of 3 diets mixed daily to follow the requirement estimate curve until 114 kg, then fed a common diet from 114 kg to market. Overall ADG was not influenced by feeding strategy, but diet blending decreased (*P* = 0.017) overall ADFI. Diet blending increased G:F (*P* = 0.019) from 26 to 114 kg, but not overall. In the experimental period, IOFC was unaffected by treatment, though feed cost per pig tended to be reduced (*P* ≤ 0.096) with blended diets. In conclusion, phase feeding and diet blending supported similar growth and carcass traits. Although IOFC was generally unaffected, diet blending reduced feed usage and feed cost/kg gain compared to phase feeding.

## Teaser

Diet blending resulted in improved feed efficiency but similar income over feed cost compared to phase fed finishing pigs.

## Introduction

Modern pig production aims to maximize animal performance while reducing nutrient excretion. An efficient way to reduce the excretion of excess nutrients is to adjust diets based on the pig’s requirements, which progressively decrease over time (NRC 2012). Traditionally, pigs during the growing-finishing period are fed in two to five dietary phases with varying standardized ileal digestible **(SID)** Lys to calorie ratios (Menegat et al. 2019). The amino acid requirements are oftentimes calculated at the middle of the weight range for each phase, and this consequently results in underfeeding the pigs for the first half of the phase and over-feeding during the second half of the phase (Menegat et al. 2020a). Increasing the number of feeding phases can reduce the degree of nutrient imbalance within each phase, but this approach also creates logistical challenges. Multiple diets require additional investment in feed formulation, manufacturing, and feed storage capacity which can complicate feed management especially in commercial settings (Pomar et al. 2021).

As an alternative to phase feeding, blending two diets at calculated proportions to meet the pigs daily estimated requirements offers the opportunity to match the daily requirements and allows more precise and efficient nutrient delivery to pigs (Pomar and Remus 2019). The ability to blend two diets has become increasingly feasible with advances in automated feeding systems that allow for accurate and flexible diet mixing at the farm level on a daily basis.

Results comparing diet blending to phase feeding on growth performance and economics are conflicting. Some studies report improvements in feed efficiency and reduced nutrient excretion (Andretta et al. 2014; Pomar et al. 2014) while others show little to no benefit compared with traditional feeding programs (Frobose et al. 2014). The different responses might be related to the number of dietary phases provided to pigs vs. the feed blended pigs. Given the variability, the present studies were conducted to compare phase feeding and diet blending on growth performance, carcass characteristics, and diet economics of finishing pigs in a commercial environment. Our hypothesis was that diet blending would offer improvements in nutrient utilization and reduce feed costs.

## Materials and methods

All protocols were approved by the Kansas State University Institutional Animal Care and Use Committee (IACUC-4847 and IACUC-4892). Experiment 1 was conducted at a commercial research-finishing site in southwest MN (Hord Farms West, Pipestone, MN) using two naturally ventilated and double-curtain-sided barns. Pens were equipped with 5-hole stainless steel dry self-feeders and bowl waterers, allowing ad libitum access to feed and water. Pens were 3.05 m × 5.49 m providing 0.62 m^2^ per pig. Experiment 2 was carried out in two barns at a commercial research-finishing site also in southwest MN (New Fashion Pork, Jackson, MN). The barns were completely mechanically ventilated with a tunnel ventilation system. Pens were fitted with 3-hole stainless steel dry self-feeders and single pan waterers, providing ad libitum access to feed and water. Pens were 2.59 × 5.56 m providing 0.76 m^2^ per pig. In both experiments, daily feed additions to each pen were accomplished using a robotic feeding system (FeedPro, FeedLogic, ComDel Innovation, Wahpeton, ND) capable of blending and recording feed amounts delivered to individual pens.

### Animals and diets

**Figure 1:**
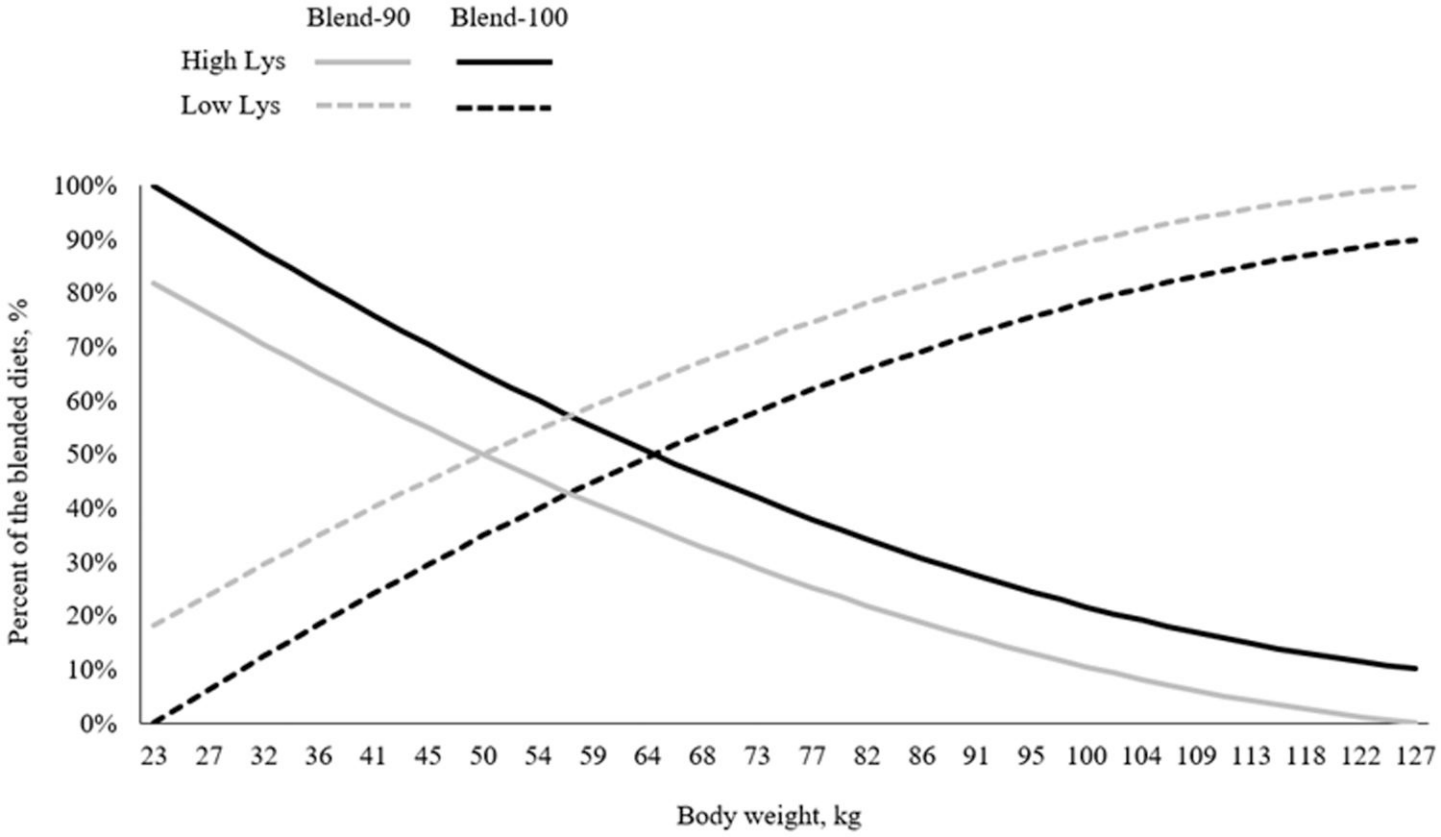
Blending of low (broken line) and high (solid line) standardized ileal digestible (SID) lys diets to achieve the targeted SID lys curves corresponding to 90% (blend-90, gray) and 100% (blend-100, black) of PIC (2022) SID lys recommendations, exp. 1. Feed was delivered daily using a robotic feeding system (FeedPro; FeedLogic by ComDel Innovation, Wahpeton, ND)

A total of 2160 pigs (PIC 337 × 1050) initially 24.8 ± 0.97 kg and 962 pigs ([Fast LW × PIC L02] × PIC 800) initially 26.5 ± 0.37 kg were used in two separate experiments. Pigs were housed in mixed gender pens with 27 pigs per pen and 20 pens per treatment in Exp. 1, and 18 to 19 pigs per pen and 26 pens per treatment in Exp. 2. Treatments were arranged in a randomized complete block design, with barn and initial body weight **(BW)** as blocking factors. In Exp. 1, pens of pigs were assigned to one of four treatments arranged in a 2 × 2 factorial with main effects of feeding strategies (phase feeding or diet blending) and SID Lys level (90 or 100% of requirement estimates; PIC 2022). Diets in the phase feeding strategies were fed from 23 to 34, 34 to 64, 64 to 88, 88 to 109, and 109 to 127 kg (marketing). Pigs were fed on a feed budget set at 21, 65, 67, 63, and 62 kg of feed per pig for phases 1 to 5, respectively. Diets for phase feeding with 100% SID Lys were formulated to contain 4.86, 4.15, 3.40, 2.96, and 2.71 g SID Lys per Mcal NE for phases 1 to 5, respectively (Table 1). Diets for phase feeding with 90% SID Lys diets were formulated to provide 90% of the SID Lys per Mcal NE levels of the Phase-100 diets. Phase changes took place when the allotted feed budgets were consumed on a barn basis. For the diet blending treatments, low and high SID Lys diets were formulated to contain 2.37 and 5.09 g SID Lys per Mcal NE (Table 2). These diets were blended daily at different proportions until the pigs reached 116 kg to meet the targeted 90 and 100% of the SID Lys curves (**Blend-90** and **Blend-100**), respectively (Fig. 1). When pigs reached 116 kg, pigs previously fed the blended diets were fed the phase 5 diet of the phase feeding strategy that corresponded to the same SID Lys level. Diet blending was implemented only up to 116 kg BW because beyond this point the blending ratios would result in one diet contributing less than 5% of the total blend which is not practical for the feed delivery system. The ratios of other essential SID amino acid (AA) to SID Lys were set to meet estimated requirements relative to Lys (PIC 2022).

**Table 1:** Diet composition of the phase-feeding treatments (as-fed basis) in Exp. 1.^a^

	90% SID Lys^b^					100% SID Lys^b^				
Items	Phase 1	Phase 2	Phase 3	Phase 4	Phase 5	Phase 1	Phase 2	Phase 3	Phase 4	Phase 5
Ingredient, %										
Corn	70.47	76.33	82.41	85.80	87.33	66.52	72.80	79.59	83.71	85.87
Soybean meal, 47.7%^c^	25.93	20.45	14.76	11.82	10.45	29.81	23.96	17.49	13.83	11.78
Limestone	1.17	1.10	1.00	0.93	0.90	1.17	1.10	1.03	0.93	0.90
Monocalcium phosphate, 21%	0.83	0.63	0.48	0.23	0.15	0.78	0.56	0.45	0.20	0.15
Salt	0.55	0.55	0.55	0.55	0.55	0.55	0.55	0.55	0.55	0.55
Liquid lysine, 55%	0.51	0.48	0.44	0.39	0.35	0.54	0.50	0.47	0.44	0.42
Thr^d^	0.19	0.16	0.13	0.10	0.08	0.22	0.18	0.14	0.12	0.12
DL-Met	0.14	0.10	0.05	0.01	0.00	0.17	0.12	0.07	0.04	0.02
L-Trp	0.02	0.02	0.02	0.02	0.02	0.02	0.02	0.02	0.02	0.02
L-Val	0.05	0.03	0.01	0.00	0.00	0.07	0.04	0.02	0.00	0.00
Tribasic copper chloride	0.03	0.03	0.03	0.03	0.03	0.03	0.03	0.03	0.03	0.03
Vitamins and trace mineral	0.10	0.10	0.10	0.10	0.10	0.10	0.10	0.10	0.10	0.10
Phytase^e^	0.05	0.05	0.05	0.05	0.05	0.05	0.05	0.05	0.05	0.05
Total	100	100	100	100	100	100	100	100	100	100
Calculated analysis										
SID amino acids, %^f^										
Lys	1.09	0.94	0.78	0.69	0.63	1.21	1.04	0.87	0.76	0.70
Ile:Lys	60	60	60	62	63	60	60	60	60	60
Met:Lys	36	35	33	30	31	37	35	34	32	31
Met and Cys:Lys	60	60	60	60	61	60	60	60	60	60
Thr:Lys	65	65	65	65	66	65	65	65	65	66
Trp:Lys	19	19	19	19	19	19	19	19	19	19
Val:Lys	70	70	70	72	75	70	70	70	70	71
Leu:Lys	129	137	148	159	169	125	132	141	150	156
His:Lys	40	41	43	45	47	39	40	42	43	44
Total Lys, %	1.22	1.06	0.88	0.78	0.72	1.35	1.17	0.97	0.86	0.79
Net energy, kcal/kg	2498	2526	2555	2575	2582	2485	2513	2544	2566	2577
SID Lys:NE, g/Mcal	4.38	3.73	3.06	2.66	2.44	4.86	4.15	3.40	2.96	2.71
Crude protein, %	18.78	16.57	14.27	13.06	12.49	20.39	18.01	15.39	13.92	13.09
Ca, %	0.70	0.62	0.54	0.45	0.43	0.70	0.62	0.55	0.46	0.43
P, %	0.55	0.48	0.42	0.36	0.33	0.55	0.48	0.43	0.36	0.34
STTD P, %^g^	0.43	0.38	0.33	0.28	0.26	0.43	0.37	0.33	0.28	0.26
Diet cost (low), US $/kg	0.214	0.198	0.181	0.170	0.165	0.226	0.208	0.189	0.177	0.171
Diet cost (high), US $/kg	0.349	0.332	0.315	0.303	0.298	0.362	0.343	0.324	0.311	0.305

a Diets in phase feeding strategies were provided from 23 to 34, 34 to 64, 64 to 88, 88 to 109, and 109 to 127 kg. Pigs in phase feeding strategies were set to receive feed budgets of 21, 65, 67, 63, and 62 kg of feed per pig for phases 1 to 5, respectively.

b SID Lys levels represent 90 or 100% of the PIC (2022) SID Lys to calorie ratio recommended for growing-finishing pigs.

c Net energy of soybean meal was assumed to be 85% of the NE of corn.

d Thr Pro (CJ America) with SID Thr of 80%.

e Optiphos Plus 2500 G (Huvepharma, St. Joseph, MO) provided 1250 FTU/kg diet with an assumed release of 0.13% STTD P.

f SID = standardized ileal digestible amino acid.

g STTD P = standardized total tract digestible P.

**Table 2:** Diet composition of the low and high SID lys diets used in diet blending treatments (as-fed basis) in exp. 1.^a^

Item	High Lys	Low Lys
Ingredient, %		
Corn	64.50	87.31
Soybean meal, 47.7%^b^	31.82	10.52
Limestone	1.18	0.90
Monocalcium phosphate, 21%	0.78	0.15
Salt	0.53	0.56
Liquid lysine, 55%	0.55	0.32
Thr^c^	0.23	0.06
DL-Met	0.19	0.00
L-Trp	0.02	0.01
L-Val	0.07	0.00
Tribasic copper chloride	0.03	0.03
Vitamins and trace mineral	0.10	0.10
Phytase^d^	0.05	0.05
Total	100	100
Calculated analysis		
SID amino acids, %^e^		
Lys	1.26	0.61
Ile:Lys	60	65
Met:Lys	37	32
Met and Cys:Lys	60	63
Thr:Lys	65	66
Trp:Lys	19	19
Val:Lys	70	78
Leu:Lys	123	174
His:Lys	39	48
Total Lys, %	1.41	0.70
Net energy, kcal/kg	2476	2582
SID Lys:NE, g/Mcal	5.09	2.37
Crude protein, %^f^	21.20	12.48
Ca, %	0.71	0.43
P, %	0.56	0.33
STTD P, %^f^	0.43	0.26
Diet cost (low), US $/kg	0.231	0.164
Diet cost (high), US $/kg	0.366	0.296

a For the diet blending strategies, diets were blended at different proportions on a daily basis to meet the targeted 90 and 100% of the SID Lys curve for curve-90% and curve-100%, respectively.

b Net energy of soybean meal was assumed to be 85% of the NE of corn.

c Thr Pro (CJ America) with SID Thr of 80%.

d Optiphos Plus 2500 G (Huvepharma, St. Joseph, MO) provided 1250 FTU/kg diet with an assumed release of 0.13% STTD P.

e SID = standardized ileal digestible amino acid.

f STTD P = standardized total tract digestible P.

**Figure 2:**
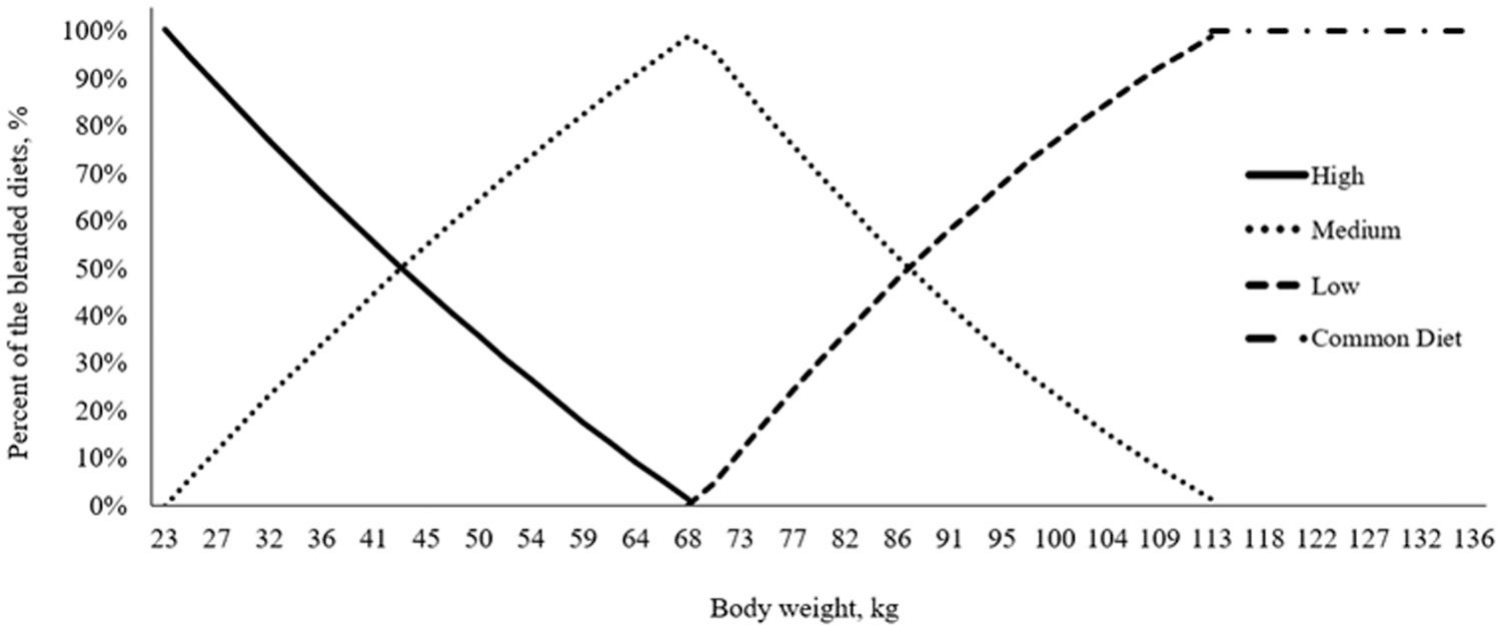
Blending of two of the three diets (high, medium, and low) to achieve the targeted standardized ileal digestible (SID) lys curve corresponding to 100% of PIC (2022) SID lys recommendations, exp. 2. Feed was delivered daily using a robotic feeding system (FeedPro; FeedLogic by ComDel Innovation, Wahpeton, ND)

In Exp. 2, pigs were assigned to one of the two feeding strategies (phase or diet blending). The experimental period spanned from 23 to 114 kg BW, with common feeding period from 114 to 135 kg BW. For the phase feeding strategy (**Phase**), diets were provided in three phases covering BW ranges of 23 to 45, 45 to 79, and 79 to 114 kg, with feed budgets of 44, 82, and 104 kg per pig, respectively. Diets were formulated to contain 4.65, 3.75, 3.00, and 2.65 g SID Lys per Mcal NE for phases 1 to 3 and the common phase, respectively. Phase changes took place when the allotted feed budgets were consumed on a barn basis. For the diet blending strategy (**Blend**), three diets were formulated with 5.09 g (High), 3.60 g (Medium), and 2.76 g (Low) SID Lys per Mcal NE, targeting lysine requirements at 23, 68, and 114 kg BW, respectively. Two of the three diets were blended daily to meet 100% of the estimated SID Lys requirement curve up to 114 kg BW (PIC 2022). From 23 to 68 kg BW, the high and medium diets were blended, whereas from 68 to 114 kg BW, the medium and low diets were blended (Fig. 2). After 114 kg BW, pigs were fed a single diet containing 2.65 g SID Lys per Mcal NE, which was the same diet fed to the phase-fed pigs from 114 to 136 kg BW. The blending was only applied up to 114 kg to accommodate slaughter plant’s requirement for a final diet without dried distillers grains with solubles. Moreover, and similar to Exp. 1, diet blending was implemented only up to 114 kg BW because, beyond this point, one diet would contribute less than 5% of the blend. Similar to Exp. 1, all diets met or exceeded requirement estimates of other essential SID amino acids as a ratio of SID Lys (Table 3).

**Table 3:** Diet composition of the phase-feeding and diet blending treatments (as-fed basis) in Exp. 2.

Item	Diets for phase feeding^a^				Diets for blending^b^		
	Phase 1	Phase 2	Phase 3	Common	High	Med	Low
Ingredient, %							
Corn^c^	61.56	69.24	75.68	87.77	58.27	70.62	77.75
Soybean meal^d^	20.21	13.05	6.96	10.16	23.19	11.73	4.98
DDGS, 6.4% oil^e^	15.00	15.00	15.00	–	15.00	15.00	15.00
Limestone	1.19	1.17	1.19	0.90	1.20	1.18	1.15
Dicalcium phosphate, 18.5%	0.54	0.20	–	0.05	0.70	0.15	–
Salt	0.44	0.45	0.46	0.55	0.43	0.45	0.46
L-Lys HCl	0.53	0.49	0.45	0.33	0.57	0.48	0.43
DL-Met	0.13	0.07	0.03	0.02	0.17	0.07	–
L-Thr	0.19	0.15	0.12	0.10	0.22	0.15	0.10
L-Trp	0.05	0.05	0.05	0.04	0.06	0.05	0.05
L-Val	0.07	0.05	0.02	0.02	0.10	0.04	–
Vitamins and trace mineral^f^	0.10	0.10	0.08	0.08	0.10	0.10	0.08
Total	100	100	100	100	100	100	100
Calculated analysis							
SID amino acids, %^g^							
Lys	1.15	0.94	0.76	0.69	1.25	0.90	0.70
Ile:Lys	56	56	56	57	56	56	56
Met:Lys	35	33	31	30	36	33	29
Met and Cys:Lys	58	58	58	58	58	58	58
Thr:Lys	65	65	65	65	65	65	65
Trp:Lys	20	20	19	20	20	20	20
Val:Lys	70	70	70	70	70	70	70
Leu:Lys	135	147	164	153	129	150	171
His:Lys	39	41	43	42	38	41	44
Total Lys, %	1.31	1.08	0.89	0.78	1.42	1.04	0.82
Net energy, kcal/kg	2474	2509	2538	2593	2458	2515	2546
SID Lys:NE, g/Mcal	4.65	3.75	3.00	2.65	5.09	3.60	2.76
CP, %	18.48	15.45	12.86	10.54	19.79	14.89	12.01
Ca, %	0.68	0.56	0.50	0.41	0.73	0.55	0.48
P, %	0.55	0.45	0.39	0.31	0.59	0.44	0.38
STTD P, %^h^	0.40	0.33	0.30	0.27	0.43	0.32	0.30
Diet cost (low), US $/kg	0.202	0.180	0.160	0.163	0.214	0.176	0.154
Diet cost (high), US $/kg	0.344	0.321	0.300	0.297	0.358	0.317	0.293

a Diets in phase feeding strategies were provided from 23 to 45, 45 to 79, and 79 to 114 kg. Pigs were fed on a feed budget set at 44, 82, and 104 kg of feed per pig for phases 1 to 3, respectively. From 114 to 135 kg, a common diet was fed until the end of the study.

b For the diet blending strategy, two of the three diets were blended at different proportions daily to meet the targeted SID Lys curve, aligned with 100% of PIC (2022) SID Lys recommendation.

c Crude protein of corn is 6.07%.

d Net energy of soybean meal was assumed to be 85% of the NE of corn and CP was 47.09%.

e Crude protein of dried distillers grain with solubles (DDGS) was 26.55%.

f Optiphos Plus 2500 G (Huvepharma, St. Joseph, MO) was included in the premix and provided 1250 FTU/kg diet with an assumed release of 0.13% STTD P.

g SID = standardized ileal digestible amino acid.

h STTD P = standardized total tract digestible P.

In both experiments, the number of pigs per pen, pen weight, and feed delivery were recorded approximately every 14 d to calculate average daily gain **(ADG)**, average daily feed intake **(ADFI)**, and gain-to-feed ratio **(G:F)**. In Exp. 1, the three heaviest pigs per pen were visually selected and marketed four weeks before the end of the study. These pigs were included in the growth performance calculations, but not carcass data. The remaining pigs were tattooed for pen identification and sent to a commercial abattoir (JBS Swift, Worthington, MN) for carcass yield, backfat, loin depth, percentage lean, and hot carcass weight measurements. In Exp. 2, four to seven of the heaviest pigs per pen were visually selected and marketed two weeks before the trial ended, with a similar number of pigs remaining in each pen. These remaining pigs were marketed at trial conclusion to a commercial abattoir (Triumph Foods, St. Joseph, MO). No carcass characteristics were collected in this experiment.

The growth performance data served as the basis for subsequent economic analyses which included calculations of feed cost, revenue, and income over feed cost **(IOFC)**. In both experiments, diet costs included the sum of formula cost, and grinding, mixing, and delivery charges. Total feed cost per pig was calculated as the product of treatment-associated diet costs and total feed intake. Feed cost per pig was calculated by dividing the total feed cost by the number of pigs placed at the start of the experiment, and feed cost per kg of gain was calculated by dividing the total feed cost by the total gain. Total revenue was calculated by multiplying the total gain per pig by carcass yield and carcass price. Because carcass traits were not measured in Exp. 2, an assumed carcass yield of 75% was used. Income over feed cost was then determined as the difference between total revenue and total feed cost. For both experiments, diet economics were determined under low and high price and cost scenarios. Ingredient and carcass prices ($/kg) under the low- and high-price scenarios, respectively, were as follows: corn, 0.110 and 0.243; soybean meal, 0.331 and 0.441; monocalcium phosphate, 0.507 and 0.618; DL-methionine, 3.748 and 5.511; L-tryptophan, 6.614 and 11.023; L-valine, 5.511 and 8.818; liquid Lys, 1.874 and 3.000; Thr Pro, 1.765 and 2.823; and carcass, 1.301 and 1.940. The cost of grinding, mixing and delivery was $0.0165/kg. Additionally for Exp. 2, diet economics were calculated separately for the experimental and overall periods.

### Statistical analysis

All data were analyzed using R (Version 4.3.1; R Core Team, Vienna, Austria) as randomized complete block designs with pen as the experimental unit. In Exp. 1, SID Lys level, feeding strategy, and the associated interaction were fixed effects, with barn and initial body weight block as random effects. One pen of pigs fed 90% SID Lys using diet blending was excluded due to a feed delivery error. For hot carcass weight (**HCW**), pig served as the observational unit, pen was included as a random effect to account for subsampling, and HCW was used as a covariate in analyses of carcass characteristics. Analyses for Exp. 2 were conducted similarly to those for Exp. 1, with the exception of treatment being the only fixed effect. Results were considered significant at *P* ≤ 0.05 and marginally significant (tendency) at 0.05 < *P* ≤ 0.10.

## Results

### Experiment 1

There were no significant interactive or main effects of feeding strategy and SID Lys observed for BW in any period of the study (Table 4). Phase fed pigs tended to have increased ADG from d 0 to 16 (*P* = 0.054) and d 94 to 108 (*P* = 0.090) compared with pigs fed blended diets. Pigs fed 90% of estimated SID Lys requirement had greater (*P* = 0.030) ADG than those fed 100% from d 58 to 72. However, overall ADG was not influenced by feeding strategy or SID Lys level. For ADFI, no interactions between feeding strategy and SID Lys level were observed for any period. Pigs fed blended diets had decreased (*P* ≤ 0.025) ADFI compared to phase feeding from d 0 to 29, d 58 to 94, and overall, whereas SID Lys level only influenced ADFI from d 0 to 16, with greater (*P* = 0.050) intake for the 90% SID Lys group. The reduced ADFI with diet blending resulted in increased (*P* < 0.001) G:F compared to phase fed pigs from d 16 to 29, d 58 to 72, d 72 to 94, and overall. On the other hand, G:F of phase-fed pigs from d 94 to 108 tended to be greater (*P* = 0.089) compared to pigs fed blended diets. Gain to feed ratio did not differ between SID Lys levels except from d 0 to 16 and d 44 to 58, when it was greater (*P* ≤ 0.041) for pigs fed diets formulated to 100% of their SID Lys requirements.

**Table 4:** Effects of phase feeding vs. complete diet blending and SID lys on finishing pig growth performance, carcass traits, and diet economics, Exp. 1.

Feeding strategy:	Phase feeding^a^		Diet blending^b^		SEM	*P* =		
SID Lys level^c^:	90%	100%	90%	100%		Strategy × Lys	Strategy	SID Lys
Growth performance								
Body weight, kg								
d 0	24.8	24.8	24.9	24.8	0.97	0.995	0.913	0.835
d 16	37.1	37.0	36.5	36.7	1.33	0.642	0.161	0.820
d 29	50.0	49.9	49.7	50.0	1.49	0.579	0.767	0.727
d 44	64.2	63.9	63.6	64.0	1.66	0.501	0.576	0.978
d 58	78.8	78.7	78.3	78.4	1.70	0.847	0.476	0.999
d 72	92.4	91.5	91.8	91.8	1.63	0.412	0.765	0.376
d 94	108.8	107.8	108.4	108.4	1.55	0.414	0.926	0.394
d 108	118.7	117.3	117.6	117.8	1.71	0.253	0.736	0.389
d 120	128.5	127.6	128.1	128.0	1.66	0.676	0.973	0.588
d 0 to 16								
ADG, kg	0.78	0.78	0.75	0.76	0.030	0.515	0.054	0.500
ADFI, kg	1.45	1.39	1.38	1.37	0.053	0.239	0.025	0.050
G:F	0.535	0.559	0.539	0.556	0.0070	0.519	0.880	<0.001
d 16 to 29								
ADG, kg	0.92	0.93	0.93	0.95	0.020	0.515	0.139	0.427
ADFI, kg	1.88	1.87	1.79	1.82	0.053	0.348	0.001	0.522
G:F	0.494	0.497	0.525	0.526	0.0110	0.764	<0.001	0.731
d 29 to 44								
ADG, kg	1.01	1.00	0.99	0.99	0.019	0.376	0.214	0.629
ADFI, kg	2.18	2.20	2.15	2.15	0.050	0.537	0.109	0.678
G:F	0.465	0.454	0.461	0.465	0.0050	0.069	0.338	0.321
d 44 to 58								
ADG, kg	1.03	1.05	1.05	1.03	0.014	0.075	0.554	0.953
ADFI, kg	2.56	2.46	2.49	2.51	0.046	0.011	0.609	0.093
G:F	0.405	0.430	0.422	0.412	0.0070	<0.001	0.828	0.041
d 58 to 72								
ADG, kg	0.96	0.92	0.96	0.94	0.024	0.540	0.464	0.030
ADFI, kg	2.69	2.67	2.59	2.58	0.042	0.858	0.003	0.666
G:F	0.356	0.345	0.371	0.364	0.0070	0.625	<0.001	0.050
d 72 to 94								
ADG, kg	0.73	0.73	0.75	0.74	0.015	0.794	0.286	0.999
ADFI, kg	2.44	2.36	2.30	2.29	0.045	0.192	<0.001	0.083
G:F	0.302	0.313	0.325	0.325	0.0050	0.126	<0.001	0.136
d 94 to 108								
ADG, kg	0.85	0.83	0.80	0.83	0.027	0.203	0.090	0.938
ADFI, kg	2.50	2.52	2.52	2.51	0.042	0.806	0.918	0.824
G:F	0.338	0.330	0.319	0.329	0.0090	0.126	0.089	0.893
d 108 to 120								
ADG, kg	0.77	0.77	0.79	0.77	0.034	0.664	0.825	0.688
ADFI, kg	2.63	2.67	2.58	2.63	0.046	0.865	0.325	0.305
G:F	0.293	0.291	0.307	0.293	0.0130	0.580	0.496	0.452
Overall								
ADG, kg	0.88	0.87	0.87	0.87	0.009	0.718	0.727	0.704
ADFI, kg	2.27	2.25	2.20	2.21	0.032	0.327	0.002	0.538
G:F	0.385	0.388	0.396	0.395	0.0030	0.243	<0.001	0.587
Carcass traits								
HCW, kg	96.7	96.9	95.8	96.1	1.06	0.930	0.178	0.685
Carcass yield, %^d^	74.61	74.83	73.99	74.32	0.351	0.867	0.074	0.384
Percentage lean, %^e^	56.99	57.07	57.16	57.36	0.201	0.658	0.094	0.303
Fat depth, mm^e^	16.45	16.19	16.16	15.83	0.334	0.849	0.111	0.145
Loin depth, mm^e^	67.87	67.16	67.56	67.82	0.383	0.135	0.598	0.495
Diet economics								
Low price								
Feed cost per pig, $^f^	46.76	49.06	46.85	49.48	0.688	0.626	0.445	<0.001
Feed cost/kg of gain, $^g^	0.47	0.49	0.47	0.49	0.004	0.221	0.505	<0.001
Total revenue, $^h^	98.40	100.52	96.25	97.37	1.707	0.754	0.102	0.312
IOFC, $^i^	51.64	51.46	49.40	47.89	1.628	0.671	0.066	0.587
High price								
Feed cost per pig, $^f^	81.18	83.85	80.41	83.53	1.196	0.703	0.352	<0.001
Feed cost/kg of gain, $^g^	0.82	0.84	0.81	0.83	0.007	0.281	0.049	<0.001
Total revenue, $^h^	146.76	149.92	143.56	145.23	2.546	0.754	0.102	0.312
IOFC, $^i^	65.59	66.07	63.11	61.70	2.452	0.683	0.144	0.842

a Diets in phase feeding strategies were fed from 23 to 34, 34 to 64, 64 to 88, 88 to 109, and 109 to 127 kg. The 90% SID Lys diets were formulated to contain 4.38, 3.73, 3.06, 2.66, and 2.44 g SID Lys per Mcal NE for phases 1 to 5, respectively. The 100% SID Lys diets were formulated to contain 4.86, 4.15, 3.40, 2.96, and 2.71 g SID Lys per Mcal NE for phases 1 to 5, respectively. Pigs in phase feeding strategies were set to receive feed budgets of 21, 65, 67, 63, and 62 kg of feed per pig for phases 1 to 5, respectively.

b For the diet blending strategies, low and high SID Lys diets were formulated to contain 2.37 and 5.09 g SID Lys per Mcal NE. These diets were blended at different proportions on a daily basis to meet the targeted 90 and 100% of the SID Lys curve, respectively.

c SID Lys levels represent 90 or 100% of the PIC (2022) SID Lys to calorie ratio recommendation for growing pigs.

d Carcass yield was calculated based on adjusted live weight. Adjusted live weight = (pen live weight—total weight of light and cull pigs)/(pen inventory—number of light and cull pigs). For phase feeding, the numbers of light and cull pigs were 31 (90% SID Lys) and 37 (100% SID Lys), whereas for the diet blending strategy, they were 27 (90% SID Lys) and 15 (100% SID Lys).

e Data were analyzed using HCW as a covariate.

f Feed cost per pig = total feed cost divided by number of pigs at placement. For phase-fed group, total feed cost was calculated using weighted feed cost of the 5 diet phases. For the diet blending group, total feed cost was calculated based on the cost of high and low Lys diets.

g Feed cost per kg of gain = total feed cost divided by the total gain.

h Total revenue = (total gain × carcass yield) × carcass price ($/kg). Total gain includes the weight of marketed, topped, and light pigs.

i Income over feed cost = total revenue—total cost.

Carcass yield tended to decrease (*P* = 0.074), and percentage lean tended to be greater (*P* = 0.094) in pigs fed blended diets than phase-fed. Conversely, HCW, fat depth, and loin depth were not affected. The SID Lys level had no effect on carcass traits.

Economic responses showed no interactions between feeding strategy and SID Lys under high- or low-price scenarios. In the low-price scenario, feeding strategy did not affect feed cost per pig or per kg gain, but phase feeding tended (*P* = 0.066) to increase IOFC. In the high-price scenario, feed cost per pig was unaffected, but diet blending reduced (*P* = 0.049) feed cost per kg gain compared with phase-fed pigs while IOFC remained unaffected. Across both scenarios, lowering SID Lys to 90% reduced (*P* < 0.001) feed cost per pig and per kilogram gain without altering total revenue or IOFC.

### Experiment 2

Feeding strategy had no effect on BW, except on d 56, when phase fed pigs had greater (*P* = 0.013) BW than pigs fed blended diets (Table 5). From d 0 to 28, ADG, ADFI and G:F were not affected by the feeding strategy. However, from d 28 to 56, pigs phase-fed had greater (*P* ≤ 0.004) ADG and ADFI but decreased (*P* = 0.020) G:F compared with diet blending. From d 56 to 84, ADG and ADFI were not influenced by the feeding strategy, but pigs fed the diet blending strategy had greater (*P* = 0.023) G:F. Over the entire experimental period (d 0 to 84), ADG was unaffected by feeding strategy, while G:F was increased (*P* = 0.019) in the diet blending group, primarily due to a reduction (*P* = 0.002) in ADFI. During the common periods (d 84 to 1116), feeding strategy did not influence ADG or ADFI. Gain to feed ratio of pigs in the phase fed group tended to increase (*P* = 0.051) compared to those in diet blending. For the overall period (d 0 to 116), diet blending resulted in lower ADFI (*P* = 0.017), but ADG and G:F were not affected.

**Table 5:** Effects of phase feeding vs. complete diet blending on growing-finishing pig growth performance and diet economics, Exp. 2.

	Feeding strategy		SEM	*P* =
Item	Phase^a^	Blend^b^		
Growth performance				
Body weight, kg				
d 0	26.5	26.5	0.37	0.721
d 28	52.1	52.1	0.57	0.764
d 56	82.2	81.2	0.60	0.013
d 84	113.1	112.5	0.68	0.189
d 102	131.7	130.9	0.70	0.153
d 116	139.5	138.9	0.84	0.456
Initial marketing (d 102)^c^	144.7	143.7	0.97	0.261
Final marketing (d 116)^d^	140.4	140.6	0.76	0.819
Average market weight^e^	141.8	141.6	0.71	0.827
d 0 to 28				
ADG, kg	0.91	0.91	0.009	0.975
ADFI, kg	1.75	1.74	0.022	0.813
G:F	0.520	0.522	0.0055	0.819
d 28 to 56				
ADG, kg	1.06	1.03	0.006	0.004
ADFI, kg	2.58	2.46	0.023	<0.001
G:F	0.412	0.421	0.0031	0.020
d 56 to 84				
ADG, kg	1.10	1.11	0.009	0.247
ADFI, kg	3.24	3.20	0.031	0.146
G:F	0.339	0.347	0.0031	0.023
Experimental period (d 0 to 84)				
ADG, kg	1.02	1.02	0.005	0.115
ADFI, kg	2.52	2.46	0.021	0.002
G:F	0.407	0.413	0.0031	0.019
Common period (d 84 to 116)				
ADG, kg	1.02	1.01	0.008	0.393
ADFI, kg	3.39	3.44	0.017	0.105
G:F	0.300	0.294	0.0022	0.051
Overall period (d 0 to 116)				
ADG, kg	1.02	1.01	0.004	0.107
ADFI, kg	2.73	2.70	0.018	0.017
G:F	0.374	0.376	0.0024	0.404
Diet economics				
Low price				
Experimental period				
Feed cost per pig, $^f^	37.32	36.65	0.411	0.096
Feed cost/kg gain, $^g^	0.443	0.439	0.0033	0.126
Total revenue, $^h^	81.95	81.31	0.679	0.433
IOFC, $/^i^	44.63	44.66	0.469	0.965
Overall period				
Feed cost per pig, $^f^	51.04	50.41	0.474	0.165
Feed cost/kg gain, $^g^	0.461	0.459	0.0031	0.628
Total revenue, $^h^	108.18	107.15	0.809	0.329
IOFC, $/^i^	57.14	56.75	0.576	0.597
High price				
Experimental period				
Feed cost per pig, $^f^	66.83	65.55	0.736	0.074
Feed cost/kg gain, $^g^	0.796	0.787	0.0057	0.074
Total revenue, $^h^	122.23	121.28	1.013	0.433
IOFC, $/^i^	55.40	55.73	0.673	0.669
Overall period				
Feed cost per pig, $^f^	91.89	90.78	0.850	0.177
Feed cost/kg gain, $^g^	0.829	0.827	0.0053	0.661
Total revenue, $^h^	161.35	159.82	1.207	0.329
IOFC, $/^i^	69.46	69.04	0.832	0.683

a Diets in phase feeding strategies were provided from 23 to 45, 45 to 79, and 79 to 114 kg. Pigs were fed on a feed budget set at 44, 82, and 104 kg of feed per pig for phases 1 to 3, respectively. From 114 to 135 kg, a common diet was fed until the end of the study. Diets were formulated to contain 4.65, 3.75, 3.00, and 2.65 g SID Lys per Mcal NE for phases 1 to 3 and the common diet, respectively.

b For the diet blending strategy, three diets were formulated to contain 5.09, 3.60, and 2.76 g SID Lys per Mcal NE. Two of these three diets were blended at different proportions daily to meet the targeted SID Lys curve [aligned with 100% of PIC (2022) SID Lys recommendation] until pigs reached 114 kg BW. Thereafter, pigs were then fed a common diet (2.65 g SID Lys per Mcal NE) from 114 to 135 kg.

c On d 102 of the trial, the four to seven heaviest pigs in each pen were selected for marketing.

d Pigs marketed on d 116 of the trial. Pigs with defects or too small for marketing were left and excluded from marketing.

e Weighted average of marketed pigs (initial and final marketing).

f Feed cost per pig = total feed cost divided by number of pigs at placement. Total feed cost was determined as the sum-product of the total feed consumed from the beginning until the end of the experimental or overall period for each diet and its corresponding cost.

g Feed cost per kg of gain = total feed cost divided by the total gain during experimental or overall period.

h Total revenue = total gain during experimental or overall period per pig × liveweight price ($/kg). For the overall period, total gains included the weight of the marketed pigs (topped and dumped).

i Income over feed cost (IOFC) = total revenue during experimental or overall period—total cost during experimental or overall period.

In the low-price scenario, feed cost per pig tended to be decreased (*P* = 0.096) with diet blending during the experimental period. Feed cost per kilogram gain, total revenue, and IOFC during the experimental period, and diet economics during the overall period were not affected by feeding strategy. In the high price scenario, pigs fed using the diet blending strategy tended to have lower (*P* = 0.074) feed cost per pig and feed cost per kilogram of gain compared to those fed the phase feeding strategy. However, total revenue and IOFC were not affected by the feeding strategy. Over the entire period, feeding strategy had no effect on feed cost per pig, feed cost per kilogram of gain, total revenue, or IOFC.

## Discussion

The goal of feeding growing pigs is to provide balanced nutrition that optimizes growth while keeping feed cost as low as possible in order to maximize profit. In growing pigs, protein accretion follows a quadratic increase as BW increases, reflecting a shift in nutrient utilization from lean tissue deposition towards fat deposition. At the same time, feed intake increases; therefore, the nutrient density of the diet is typically reduced as pigs become heavier. To account for the dynamic changes in nutrient requirements, feeding programs are often designed to adjust diet composition throughout the growing period. The most common feeding strategy is phase feeding where multiple diets are fed to a group of pigs based on budget or weight ranges to closely meet the nutrient requirements (Menegat et al. 2020a). In phase feeding strategies, nutrient requirements are typically set to the mid-point of the pig’s weight range, resulting in nutrient levels fed below the requirements initially and then nutrients will exceed the requirements toward the end of the phase. Although increasing the number of feeding phases can reduce nutrient imbalances because the nutrient composition of each diet is closer to the pigs’ biological requirements at both the beginning and end of each phase, it may also introduce greater complexity in feed management and require additional investment in feed processing and storage. An alternative program is curve or diet blending where two basal diets are blended in calculated proportions to more closely match the pigs’ daily nutrient requirements (Frobose et al. 2014). This approach has become more feasible with the adoption of automated feeding systems. Diet blending also offers an opportunity to decrease nutrient excretion without affecting performance. Andretta et al. (2016) and Llorens et al. (2024) observed that daily blending of diets allows for a reduction in daily N and P excretion by more than 30 and 40%, respectively. Precisely meeting the nutrient requirements allows for a decrease in nutrient excretion and feed cost (Ferreira et al. 2024).

While studies have compared phase feeding and diet blending, results for growth performance and economics have been inconsistent. In Exp. 1, phase feeding was compared with blending of low and high SID Lys diets at 90 and 100% of PIC (2022) recommendations to evaluate the interaction between feeding strategy and SID Lys level. Previous evidence suggests that phase feeding may reduce performance when diets are formulated below requirements, particularly when fewer dietary phases are used and Lys levels are slightly underestimated (Menegat et al. 2020a). In such cases, growth can be compromised if initial BW or feed intake is lower than expected (Menegat et al. 2020a). On the other hand, Exp. 2 was designed to provide a more precise nutrient supply by formulating three diets, of which two were blended at any one time. In contrast to Exp. 1, this approach aimed to avoid nutrient imbalances that could arise from relying on only two extreme diets. In Exp. 1, the low SID Lys diet used in the blending strategy was formulated based on 90% of the SID Lys requirement of 127 kg pigs while the high SID Lys diet was formulated based on 100% of the SID Lys requirement of 23 kg pigs. Because blending was based solely on SID Lys, the use of these extreme diets resulted in other essential amino acid to Lys ratios being greater than those achieved with phase feeding. Nonetheless, all essential amino acid requirement estimates were met.

In both studies, final BW and overall ADG were unaffected by feeding strategy indicating that both strategies can support optimal growth. These findings are consistent with the earlier findings of Andretta et al. (2014) and Frobose et al. (2014). In contrast, Pomar et al. (2014) observed greater final BW in pigs fed blended diets, primarily due to improved ADG during the early growth period. The 3-phase program used in that study may have compromised growth during the initial phase when pigs were fed diets below their requirements for a longer period of time. Feeding fewer phases increases the likelihood of a mismatch between nutrient supply and pig requirements, thereby limiting growth (Menegat et al. 2020a).

A key finding across both of the present experiments was the reduction in ADFI of pigs fed blended diets. This decrease in feed intake improved G:F (overall in Exp. 1 and during the experimental period in Exp. 2) without compromising ADG. These observations are consistent with the findings of Lee et al. (2000) and Frobose et al. (2014) suggesting that pigs were able to maintain growth with lower intake, likely due to improved nutrient utilization. Precisely matching nutrient supply to the pigs’ requirements enhances utilization efficiency by minimizing inevitable nutrient losses and reducing nutrient excretion (Pomar and Remus 2019; Gaillard et al. 2020). Additionally, meeting the amino acid requirements more precisely eliminates the time that pigs are fed below their requirements as in phase feeding which also leads to improved efficiency.

In Exp. 1, growth performance was compared between two SID Lys levels, and results showed no differences for final BW, overall ADG, ADFI, or G:F. This aligns with the earlier findings that slight reduction in amino acid density may not compromise performance particularly in high feed intake situations (Song et al. 2022). In the present study (Exp. 1), pigs fed the diet containing 90% SID Lys had an SID Lys intake of approximately 20.7 g/kg gain. This value falls within the range of 19.6 to 23.0 g/kg gain in pigs weighing 35 to 110 kg (Main et al. 2008; Shelton et al. 2011) and the range of 17.0 to 22.6 g/kg gain in 100 to 130 kg pigs (Main et al. 2008; Soto et al. 2019) that is required to maximize growth. This indicates that the feed intake and SID Lys intake of pigs fed the 90% SID Lys diet in the present study was sufficient to support growth performance with no advantage to further increasing SID Lys.

In Exp. 2, the comparison of feeding strategies was applied only until pigs reached 114 kg, after which all pigs were fed a common diet until slaughter at 141 kg. This was necessary to meet the slaughter plant’s requirement for a DDGS-free diet during the final finishing phase. The use of a common diet, reduced the G:F advantage observed with diet blending and resulted in no overall difference in G:F.

Carcass traits were also evaluated in Exp. 1. Overall, feeding strategy did not significantly affect carcass characteristics, consistent with the findings of Moore et al. (2013, 2016) and Frobose et al. (2014), who likewise reported no differences in carcass traits between pigs fed with a phase feeding strategy or utilizing blended diets. In the present study, pigs fed blended diets showed a tendency for lower carcass yield and higher lean percentage. It should be noted that HCW and carcass yield were calculated only from pigs that were marketed and slaughtered, and numerically more pigs in the phase feeding group were not marketed compared with the diet blending group because they were either too light or had physical defects (footnote, Table 4). Similar to growth performance, the comparable carcass traits observed between the SID Lys levels can be attributed to the adequate SID Lys intake observed at both SID Lys levels.

The hypothesis that diet blending would reduce excess nutrient intake and thereby lower feed costs was supported by these results. In Exp. 1, pigs fed blended diets had lower feed cost per kilogram of gain under the high-price scenario. In Exp. 2, diet blending tended to reduce feed cost per pig during the experimental period at the low-price scenario, and both feed cost per pig and per kilogram of gain at the high-price scenario. Similarly, Ferreira et al. (2024) simulated a 2.4% reduction in feed cost with diet blending, and in the present study, feed cost per kilogram of gain was 1.0% lower for diet blending compared with phase feeding. Despite these reductions, IOFC did not differ between the two feeding strategies in both experiments. However, IOFC tended to be greater for phase fed pigs at the low-price scenario in Exp. 1. The IOFC response was driven by the numerically higher total revenue observed for the phase-fed pigs. Frobose et al. (2014) also observed a similar IOFC between phase feeding and diet blending and explained that it was due to lower HCW in pigs fed blended diets. Additionally in Exp. 1, the costs per kilogram of diet was higher for the blended diets than the weighted cost of the 5-phase diets, likely due to excess nutrients required when only using two diets to meet the requirements over a wide weight range. This partly explained the numerically higher IOFC for the phase-fed pigs compared to pigs fed blended diets. By contrast, in Exp. 2, refinement of nutrient supply by blending two of three diets minimized this difference in feed cost per kilogram diet, resulting in more comparable IOFC values between the two strategies. It is worth noting that the comparison of diet economics between feeding strategies in this study did not account for the fixed cost of the feeding system unit, its installation, or its maintenance. In addition, feed milling considerations, such as the potential benefits of mixing and managing fewer diet types, were not included. This information is needed to evaluate the total economics of feeding strategies in a pork ecosystem. Lastly, the environmental benefit of diet blending is an important factor to consider when evaluating sustainability of pork production (Navales et al. 2025). For the diet economics of SID Lys in Exp. 1, the presented study showed that the 90% SID Lys treatment had a lower feed cost per pig and per kilogram gain than pigs fed 100% of the estimated requirements. This is driven by the lower feed cost of formulating to a lower SID Lys level and this is consistent with observations of Cordoba et al. (2024).

Overall, the results of these experiments showed that both phase feeding and diet blending strategies can support optimal growth performance and carcass characteristics. Diet blending offers the opportunity to reduce feed cost per kilogram gain, especially under high feed cost scenarios. While IOFC was generally similar between feeding strategies, refinement of diet formulation can help manage feed cost and maximize feed efficiency as demonstrated in Exp. 2. Moreover, the observed similar performance and lower feed cost of the 90% SID Lys treatments highlights that the marginal reduction in AA density can be economically beneficial when feed intake is sufficient to meet requirements. The findings in the current study suggest that producers can utilize diet blending and adjust SID Lys levels as tools to improve diet economics and potentially reduce nutrient excretion while maintaining animal performance and carcass traits.

## List of abbreviations

AA: amino acid
ADFI: average daily feed intake
ADG: average daily gain
BW: body weight
CP: crude protein
G: F: gain to feed ratio
HCW: hot carcass weight
IOFC: income over feed cost
NE: net energy
SID: standardized ileal digestible
STTD: standardized total tract digestible
